# Absent Toll-like receptor-9 expression predicts poor prognosis in renal cell carcinoma

**DOI:** 10.1186/1756-9966-30-84

**Published:** 2011-09-19

**Authors:** Hanna Ronkainen, Pasi Hirvikoski, Saila Kauppila, Katri S Vuopala, Timo K Paavonen, Katri S Selander, Markku H Vaarala

**Affiliations:** 1 Department of Surgery, Oulu University Hospital, PO Box 21, 90029 OYS, Finland; 2 Department of Pathology, Lansi Pohja Central Hospital, Kauppakatu 25, 94100 Kemi, Finland; 3 Department of Pathology, University of Oulu, PO Box 50, 90014 University of Oulu, Finland; 4 Department of Pathology, Lapland Central Hospital, PO Box 8041, 96101 Rovaniemi, Finland; 5 Department of Pathology, University of Tampere and Tampere University Hospital, School of Medicine, 33014 University of Tampere, Finland; 6 Department of Medicine, Division of Hematology-Oncology, University of Alabama at Birmingham, Birmingham, AL 35294-2182, USA; 7 Department of Anatomy and Cell Biology, University of Oulu, PO Box 5000, 90014 University of Oulu, Finland

**Keywords:** renal cell carcinoma, toll-like receptor 9, tumour necrosis, prognosis

## Abstract

**Background:**

Toll-like receptor 9 (TLR9) is a cellular DNA-receptor whose activation with cognate ligands triggers an immune reaction, with increased production of inflammatory cytokines. The aim of this study was to examine the expression of TLR9 in renal cell carcinoma (RCC), which is generally renowned of its immunogenic nature. We also evaluated the prognostic value of TLR9 in RCC.

**Methods:**

TLR9 expression in RCC was characterized with immunohistochemistry in a retrospective study population of 152 RCC patients who underwent renal surgery. The TLR9 staining intensity was compared with clinical parameters.

**Results:**

Of the studied tumours, 112 (81%) exhibited cytoplasmic TLR9 immunostaining. No association was detected between cytoplasmic TLR9 immunoexpression intensity and stage, nuclear grade, histological subtype or tumour necrosis. Cytoplasmic TLR9 immunoexpression was, however, a marker of favourable RCC specific survival both in univariate analysis and in multivariate regression model.

**Conclusions:**

We conclude that TLR9 expression is an independent prognostic marker of RCC and the absence of TLR9 expression is related to poorer prognosis in RCC.

## Background

Renal cell carcinoma (RCC) is a cancer of increasing incidence and mortality [1]. At the time of the diagnosis, up to one third of the patients have metastasized disease and a half of the remaining patients will experience a recurrence after an initially curative treatment [2]. Despite the many well-known prognostic factors for the disease, the behaviour of RCC is very difficult to predict.

Toll-like receptors (TLRs) are pattern recognition receptors that detect both microbe- and host-derived molecular patterns. Thus far, at least 13 mammalian TLRs have been recognized, each of them responding to a different ligand. The subcellular expression sites of the various TLRs also vary. TLRs 1, 2 and 4 are expressed and bind their ligands on the cell surface while the TLR9 subfamily (including TLRs 3, 7, 9 and 13) reside in intracellular vesicles. Ligand binding to TLRs activates transcription factors, such as NF-kappaB and the eventual outcome of TLR activation is an immune reaction, characterized by increased production of inflammatory mediators. Specifically, TLR9 is a receptor for both microbial and vertebrate DNA. The intracellular expression of TLR9 and also possibly the other endosomal TLRs is thought to evade self-recognition of DNA and RNA [3-7].

It is now well established that TLR9 is also expressed in various cancer cells, including breast, brain, ovarian, gastric, lung and prostate cancer cells [8-11]. Furthermore, in clinical breast, ovarian and prostate cancer specimens, increased TLR9 expression was associated with decreased tumour differentiation [10-13]. It has also been demonstrated that stimulation of TLR9-expressing cancer cells with synthetic TLR9-ligands increases their in vitro invasion which is associated with the down-regulation of tissue inhibitor of metalloproteinases-3 (TIMP3) and the up-regulation of matrix metalloproteinase-13 (MMP-13) activity. Although bacterial DNA, similar to the synthetic CpG-sequence containing TLR9-ligands, also induces invasion in TLR9 expressing cancer cells in vitro, the natural TLR9-ligand that might induce invasion for example in breast cancers, remains unknown [10,11].

In the normal kidney, TLR9 expression has been detected in the renal tubules and interstitial tissue, while the tubulointerstitial and glomerular expression has been detected in lupus nephritis [14]. Previously, TLR9 has been associated with renal disease, such as glomerulonephritis [15] and lupus nephritis [16]. To our knowledge, there are no previous studies of TLR9 expression in RCC. However, the efficacy of a synthetic TLR9-agonist has been studied in a clinical trial in advanced metastatic RCC. This compound was found to have only modest antitumour activity [17].

The aim of this study was to investigate TLR9 expression in RCCs and to evaluate the prognostic significance of TLR9 immunostaining in RCCs.

## Material and methods

### Patients

This retrospective clinical cohort consisted of 152 patients with 77 (51%) females and 75 (49%) males who underwent surgery for primary renal cell carcinoma between the years 1990 and 1999, at the Oulu University Hospital. All clinical data and patient follow-up details were collected from patient records and re-evaluated by the same urologist (HR). Seven patients (5%) were operated by resection and 145 (95%) by radical nephrectomy. At the time of the diagnosis, the median age of the patients was 63 years old (range 29-86 years) and the mean age was 62 (SD ± 11 years). The median and mean follow-up times were 90 (range 0-209) months and 90 (SD ± 63) months, respectively. Complete information was obtained from all patients. During the follow-up period, 44 (29%) patients died of RCC, 40 (26%) died of other causes and 68 (45%) were still alive. The distribution of the clinicopathological parameters of the tumours has been previously described [18,19]. Of the patients, 6 (4%) had lymph node metastases and 18 (12%) had distant metastases. The stage of the tumours was assigned using the TNM staging of RCC [20]. T and N classes were determined by the pathological evaluation of primary tumour and resected lymph nodes. Further, N class and M class were assessed by radiological evaluation performed before primary operation. The abdominal ultrasound was done for every patient and in addition, abdominal computed tomography (CT) was performed for 125 patients (82%). Chest radiography (X-ray and/or CT) was done for 135 patients (89%). In a case of suspected metastases or vena caval involvement, additional studies such as bone scintigraphy (14 patients, 9%), skeletal radiography (17 patients, 11%), magnetic resonance imaging (MRI) (11 patients, 7%) or cavography (3 patients, 2%) were performed. The study was approved by the local ethical board.

### Tumour samples and TLR9 immunostaining

The tumour samples were routinely fixed in 10% buffered formalin and embedded in paraffin. The histological diagnosis was confirmed by reviewing the haematoxylin and eosin (H & E) stained original sections simultaneously by two pathologists. The tumours were re-classified and graded according to the WHO classification [21]. The most representative block of the tumour was selected and cut into 3 μm thick sections, into multi-tissue blocks which were mounted onto precoated slides. Tissue sections were then deparaffinized in xylene, rehydrated in descending ethanol series and washed in phosphate buffered saline (PBS). Expression of TLR9 was analyzed by using a mouse monoclonal anti-human TLR9/CD289 (Img-305A, clone 26C593.2, Imgenex, San Diego, California, USA, dilution 1:200) antibody, as previously shown by us [13,22]. In order to enhance the immunoreactivity, the sections were incubated in a Tris-EDTA buffer (pH 9.0) and boiled. Endogenous peroxidase activity was eliminated by incubation in hydrogen peroxide and absolute methanol. The bound antibodies were visualized using Envision Detection System (K500711; Dako Denmark A/S). DAB (diaminobenzidine) was used as a chromogen. A multitissue block containing breast cancer samples and normal cervical tissue was used as a positive control.

### Scoring of TLR9 immunoreactivity

Cytoplasmic TLR9 immunoreactivity was initially scored according to four cytoplasmic staining intensities: negative (0), weak (1), moderate (2) or strong (3) [13,22]. For further statistical analyses, the negative samples (score 0) were compared with the positive ones (scores 1 to 3). Immunohistochemical staining was evaluated simultaneously by two observers (PH and MHV) who were blinded to the clinical data and a consensus on the staining intensity was reached.

### Statistical analyses

The software SPSS for Windows 15 (Chicago, IL) was used for statistical analyses. Associations between factors, including clinicopathological variables and TLR9 immunostaining patterns, were assessed by the χ2 test, or the Fisher's exact test in the case of low expected frequencies. Survival rates were calculated using the Kaplan-Meier method and the statistical significance between groups was analysed using the log-rank test. Hazard ratio (HR) was assessed by Cox univariate analysis. Renal cell carcinoma-specific survival was calculated from the date of diagnosis to death from RCC or the last day of follow-up. Deaths due to intercurrent causes were censored. Multivariate survival analysis was done with the Cox proportional hazards model; the following covariates were entered: gender, age, stage, Fuhrman grade and TLR9 immunoreactivity. All p-values were two sided.

## Results

### TLR9 protein expression in RCC

**Figure 1:**
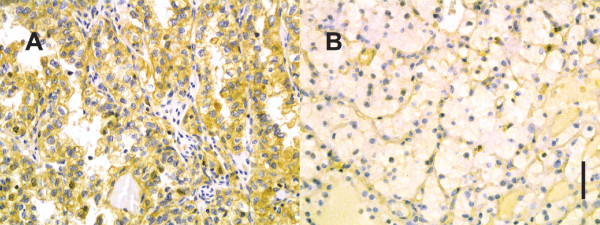
**TLR9 immunostaining in RCC**. Tumours with high cytoplasmic expression (A) and negative cytoplasmic expression (B) are shown. Magnification ×400, scale bar 50 μm.

There were 138 RCC tumours available for the evaluation of TLR9 immunoreactivity. Examples of TLR9 staining patterns are shown in Figure 1. Twenty-one (15%) of the tumours were strongly positive, 39 (28%) moderately positive, 52 (38%) weakly positive and 26 (19%) negative for cytoplasmic TLR9 immunostaining. For the further analyses, the weakly, moderately and strongly positive cases were combined and grouped as TLR9 positive samples (n = 112, 81%). Some nuclear TLR9 immunopositivity was also detected in 60 (44%) tumour samples. In addition to immunoexpression of TLR9 in the tumour cells, immunoreactivity was observed in endothelial and inflammatory cells as well as in some fibroblasts.

### Association of cytoplasmic TLR9 expression with the clinicopathological characteristics

The distributions of pT-class, stage, nuclear grade and histological subtype of RCC and their associations with cytoplasmic TLR9 expression are presented in Table 1. No statistically significant associations were detected between cytoplasmic TLR9 expression and pT-class, stage or grade. The immunoexpression of TLR9 did not associate with tumour necrosis (data not shown). There was no association between TLR9 expression and histological subtype. The immunoexpression of TLR9 was common in every histological subtype of RCC and immunopositivity for TLR9 was detected in 100 (82%), 6 (67%), 4 (80%) and 2 (100%) cases tumours representing the histological subtypes of clear cell RCC, papillary RCC, chromophobe RCC and unclassified RCC, respectively. Nuclear TLR9 expression did not have any association with these characteristics (data not shown).

**Table 1:** Associations between cytoplasmic TLR9 expression and tumour pT-class, stage, grade and histological subtype

	Cytoplasmic TLR9 expression		
	negative	positive	p-value
pT class			
pT1	12 (18%)	56 (82%)	0.31
pT2	4 (36%)	7 (64%)	
pT3	8 (15%)	45 (85%)	
pT4	2 (33%)	4 (67%)	
			
Stage			
I	11 (17%)	52 (83%)	0.27
II	4 (36%)	7 (64%)	
III	6 (13%)	39 (87%)	
IV	5 (26%)	14 (74%)	
			
Nuclear Grade			
I	0 (0%)	5 (100%)	0.69
II	13 (18%)	60 (82%)	
III	9 (25%)	27 (75%)	
IV	4 (18%)	18 (82%)	
			
Histology			
clear cell	22 (18%)	100 (82%)	0.69
papillary	3 (33%)	6 (67%)	
chromophobic	1 (20%)	4 (80%)	
undifferentiated	0 (0%)	2 (100%)	

### Prognostic significance of TLR9 expression in RCC

**Figure 2:**
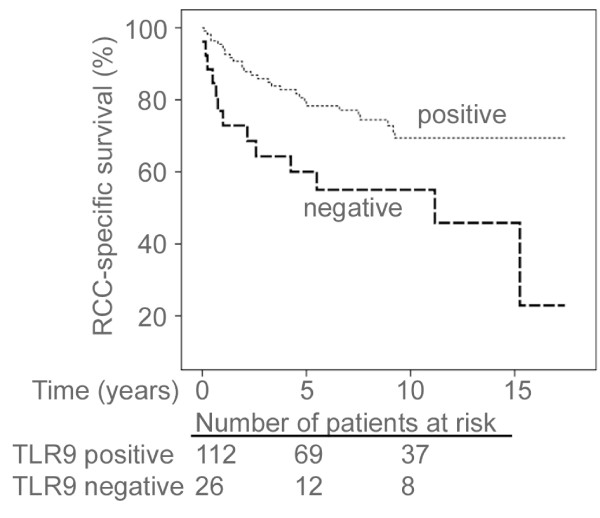
**Associations between cytoplasmic TLR9 expression and RCC-specific survival**. Patients with TLR9 negative tumours showed reduced survival when compared to patients with tumours positive for these proteins. p = 0.007

The RCC-specific survival was significantly longer for patients whose tumours did express cytoplasmic TLR9, as compared with patients whose tumours were negative for cytoplasmic TLR9 expression (p = 0.007)(Figure 2.). The hazard ratio (HR) of patients without TLR9-expressing tumours was 2.40 (95% CI 1.24-4.63, p = 0.009). The mean RCC-specific survival times for TLR9 negative and TLR9 positive tumours were 112 (95% CI 76-147) and 160 (95% CI 144-175) months, respectively (p = 0.007)

In the Cox regression analysis for cytoplasmic TLR9 expression, gender, age, stage and nuclear grade, the statistically significant factors in RCC-specific survival were stage and TLR9 expression (Table 2).

**Table 2:** Cox multivariate survival analysis in 136 patients with RCC

Covariate	Hazard ratio	95.0% CI	p-value
Male gender	0.76	0.45-1.80	0.76
Age	1.02	0.98-1.06	0.34
Stage I	1 (ref.)		
Stage II	3.03	0.89-10.3	0.076
Stage III	3.17	1.20-8.35	0.020
Stage IV	19.3	6.86-54.5	< 0.001
Fuhrman grade I or II	1 (ref.)		
Fuhrman grade III	1.13	0.49-2.57	0.78
Fuhrman grade IV	2.68	1.20-5.98	0.16
Positive cytoplasmic TLR9 expression	0.28	0.14-0.58	0.001

## Discussion

We demonstrate here for the first time that TLR9 is frequently expressed in RCCs. Although there was no association between the immunoexpression of TLR9 and histological subtype, stage or grade of RCC, cytoplasmic TLR9 expression was a statistically significant prognostic factor in RCC specific survival in both univariate and multivariate analyses and TLR9 expression was an independent marker of better prognosis in RCC. Our findings thus suggest that the lack of TLR9 confers aggressive behaviour of renal carcinoma cells. The significance of nuclear TLR9 expression remains obscure, but it may also represent unspecific staining.

Expression of TLR9 has been previously detected in various cancer cell lines and in various clinical cancer specimens. Synthetic TLR9-ligands induce cancer cell invasion *in vitro *and high TLR9 expression has been associated with poor differentiation of various cancers, suggesting that high TLR9 expression or naturally existing DNA-ligands might induce TLR9-mediated invasion, and thus contribute to worse outcomes in cancers with higher TLR9 expression. In this light, our finding demonstrating the lack of TLR9 expression as a poor prognosis marker is RCC is surprising. So far, the association between TLR9 and clinopathological parameters and the survival of cancer patient has been evaluated in only a few studies. In breast cancer it has been demonstrated that immunoexpression of TLR9 is significantly increased in high-grade tumours compared with lower-grade tumours [12,22]. Similarly, it has been shown that recurrent breast carcinomas exhibit a significant increase in the mRNA levels of TLR9 in cancer cells [23]. However, a remarkable percentage (57.5%) of recurrent breast tumours was shown to express TLR9 by fibroblast-like cells and these tumours have reported to have low probability of metastasis [23]. It has also been demonstrated that cell surface stimulation of TLR9 promotes cell proliferation and survival in hepatocellular carcinoma [24]. In neuroblastoma, TLR9 expression has been found to correlate inversely with disease stage [25] whereas in glioma, TLR9 expression has shown to be significantly higher in high grade tumours compared to low-grade gliomas and TLR9 immunoexpression has been reported to be a statistically significant marker of poorer prognosis in glioma [26]. Thus, the contribution of either high or low TLR9 expression to the pathophysiology of cancer may be highly tumour specific.

Upon the recognition of DNA, TLR9 recruits specific intracellular adaptor proteins to initiate signalling pathways and the eventual outcome is an immune reaction characterized by the increased production of inflammatory mediators like interferon and other inflammatory cytokines [3,27]. RCC is generally renowned of its immunogenic nature. RCC can allure different effector cells of both the innate and adaptive immune system including natural killer (NK) cells, dendritic cells (DC) and various T cells [28]. A variety of tumour-associated antigens (TAAs) which can evoke tumour-specific T-cell-defined immune responses in cancer patients has been detected in RCC tumours [29]. More importantly, immunotherapy with interferon alpha (IFN-α) or interleukin 2 (IL-2) can produce even complete and durable response in advanced RCC [30] and tumour vaccines have shown to have some response, too [31]. Rare cases of spontaneous regression of metastases in RCC caused probably by immunologic mechanism have been reported [32]. Thus, the prognostic significance of TLR9 expression in RCC may be associated with immune responses to the tumour cells. Hypothetically, in the absence of RCC TLR9 expression, such responses are not evoked and they are less susceptible to immunosurveillance and they can progress. These issues warrant further investigation.

Low oxygen environments can be created by various pathophysiological conditions, including infection, inflammation, tissue injury, and solid tumours [33]. Hypoxia is one of the significant features of solid tumours, including kidney tumours. Hypoxia and the compensatory hyperactivation of angiogenesis are thought to be particularly important in RCC [34]. In hypoxia, an increased expression of various TLRs including TLR9 has been demonstrated [35,36] and this induction of TLRs has shown to be coordinated by the hypoxia inducible factor 1 (HIF-1) [35]. Whether or not the absence of TLR9 in RCC is regulated by hypoxia and HIF-1 and thereby, increase the aggressive behaviour of the tumour cells also warrant further investigation.

## Conclusions

In conclusion, TLR9 immunoexpression is common in RCC, where it is associated with better prognosis in RCC and the lack of TLR9 expression in RCC predicts short survival. The favourable influence of TLR9 expression on the course of the disease may be based on the immunologic response generated to the renal carcinoma cells. The prognostic significance of TLR9 expression in RCC should be evaluated in other RCC cohorts.

## Competing interests

The authors declare that they have no competing interests.

## Authors' contributions

HR performed statistical analyses and drafted the manucript. PH evaluated the immunohistochemical staining. SK revised the manuscript. KSV carried out immunohistochemical studies. TKP conceived of the study. KSS revised the manuscript. MHV participated in the design of the study, evaluated the immunohistochemical staining and revised the manuscript. All authors read and approved the final manuscript.

## Authors' Information

Katri S Selander and Markku H Vaarala shared last authorship on this manuscript.
